# *Mycobacterium tuberculosis* Beijing Genotype and Risk for Treatment Failure and Relapse, Vietnam

**DOI:** 10.3201/eid0912.030169

**Published:** 2003-12

**Authors:** Nguyen Thi Ngoc Lan, Hoang Thi Kim Lien, Le B. Tung, Martien W. Borgdorff, Kristin Kremer, Dick van Soolingen

**Affiliations:** * Pham Ngoc Thach Tuberculosis and Lung Diseases Centre, Ho Chi Minh City, Vietnam; † Royal Netherlands Tuberculosis Association, The Hague, the Netherlands; ‡ University of Amsterdam, the Netherlands; § National Institute of Public Health and the Environment, Bilthoven, the Netherlands

**Keywords:** *Mycobacterium tuberculosis*, epidemiology, multidrug resistance, treatment failure, Beijing genotype

## Abstract

Among 2,901 new smear-positive tuberculosis cases in Ho Chi Minh City, Vietnam, 40 cases of treatment failure and 39 relapsing cases were diagnosed. All initial and follow-up *Mycobacterium tuberculosis* isolates of these case-patients had (nearly) identical restriction fragment length polymorphism patterns, and the Beijing genotype was a significant risk factor for treatment failure and relapse (odds ratio 2.8, 95% confidence interval 1.5 to 5.2).

The Beijing genotype is widespread in Asia (*1*–*3*), and has been involved in outbreaks of multidrug-resistant tuberculosis in various parts of the world, including Cuba, Germany, Russia, and Estonia (*4*–*7*). The W strain, which caused a large outbreak of multidrug-resistant tuberculosis in the United States, is a variant of the Beijing genotype (*8*–*10*). The Beijing genotype is emerging in Vietnam in association with drug resistance in this region (*11*).

In a recent study on acquired drug resistance in Ho Chi Minh City, Vietnam, drug resistance at time of enrollment in the study was shown to be an important risk factor for treatment failure and for relapse of tuberculosis after treatment was completed successfully (*12*). We used the materials collected for this study to determine the extent to which the Beijing genotype is a risk factor for treatment failure or relapse.

The methods of this study have been described previously (*12*). In brief, 2,901 new case-patients with smear results positive for *Mycobacterium tuberculosis* were enrolled in Ho Chi Minh City, Vietnam, from August 1996 through July 1998. After a case was diagnosed at the district tuberculosis center, a sputum sample from the case-patient was sent to the reference laboratory, for a repeat microscopy examination of the sputum smear to confirm the diagnosis and to be stored at –20°C. All patients received the standard regimen of the National Tuberculosis Program, i.e., 2 months of streptomycin, isoniazid, rifampicin, and pyrazinamide, followed by 6 months of isoniazid and ethambutol (2SHRZ/6HE). When treatment failure (defined as a positive sputum smear 5 or 8 months after the onset of treatment) or relapse (defined as a positive sputum smear within 2 years after scheduled treatment cessation) was noted, another sputum sample was collected, and both samples were cultured and tested for drug susceptibility with the proportion method. Restriction fragment length polymorphism (RFLP) typing was performed by using insertion element IS*6110* as a probe (*13*,*14*) to exclude reinfection and laboratory cross-contamination.

A random sample of sputum samples was collected at enrollment for culture and sensitivity testing from 10% of patients who had not experienced treatment failure or relapse (controls). This sample size would allow approximately two controls per case-patient. We performed spoligotyping on the sputum samples of case-patients who had experienced treatment failure or relapse and controls to identify the samples that belonged to the Beijing genotype (*15*). The Beijing genotype was defined as strains without spacers 1–34 and the presence of (at least 3) the spacers 35–43 (*16*).

Over the enrollment period, 6,113 new smear-positive tuberculosis patients began a treatment regimen, 2,901 of whom were included in the study. Slightly more men were enrolled than women (age-adjusted odds ratio [OR] 1.2, 95% confidence interval [CI] 1.0 to 1.3), and enrollment was particularly low in those >65 years of age (sex-adjusted OR 0.3, 95% CI 0.2 to 0.4). Of the 2,901 enrolled patients, 2,568 (88%) recovered, and 12 (0.4%) completed treatment; in 125 (4.3%), treatment failed; 63 (2.2%) died; 53 (1.8%) were transferred out; and 80 (2.8%) did not complete the study. Through December 1999, a total of 168 case-patients who experienced a relapse (6.5% of those cured or with treatment completed) were identified. Forty of 125 case-patients whose treatment failed and 39 of 168 case-patients who had a relapse had two positive cultures with nearly identical RFLP patterns (*12*). Spoligotyping results were available for 136 controls.

Case-patients were somewhat less likely than controls to be female and tended to be somewhat older than controls. However, these differences were not significant. Primary drug resistance (in comparison with full susceptibility) was a strong risk factor for treatment failure or relapse with combined ORs of 3.4 for streptomycin monoresistance, 4.2 for isoniazid monoresistance, and 23 for other susceptibility patterns (Table). The Beijing genotype was associated with treatment failure (OR 3.3 95% CI 1.3 to 8.3; p < 0.01) and relapse (OR 2.4 95% CI 1.0 to 5.7; p < 0.05). In view of the small numbers and similar odds ratios, these two groups were combined (OR 2.8, 95% CI 1.5 to 5.2) (Table). The association between the Beijing genotype and treatment failure or relapse hardly changed when taking into account primary drug resistance, age, and sex (OR 3.2, 95% CI 1.4 to 7.1). We conclude that the Beijing genotype is a risk factor for treatment failure and relapse in Vietnam, irrespective of primary drug resistance. This finding suggests that infections with Beijing genotype strains are more persistent than infections with other *M. tuberculosis* strains, which may explain the emergence of Beijing genotype strains in this region (*11*).

**Table:** Characteristics at enrollment of case-patients who experienced treatment failure and relapse and of controls who did not experience treatment failure, relapse, or die^a^

Characteristic	Failure	Relapse	All case-patients	Controls	Crude		Adjusted^b^		Adjusted^c^	
					OR	95% CI	OR	95% CI	OR	95% CI
Sex										
Male	28	36	64	104	1				1	
Female	12	3	15	39	0.63	0.32 to 1.22			0.55	0.22 to 1.38
Age group (y)										
15–34	15	9	24	60	1				1	
35–54	22	25	47	72	1.63	0.90 to 3.0			2.0	0.90 to 4.6
>55	3	5	8	11	1.82	0.65 to 5.1			2.4	0.60 to 9.9
Genotype										
Beijing	32	29	61	75	2.8	1.48 to 5.15	2.5	1.2 to 5.2	3.2	1.4 to 7.1
Other	8	10	18	61	1		1		1	
Unknown				7						
Resistance pattern										
Fully susc.	4	13	17	101	1		1		1	
S only	3	9	12	21	3.4	1.41 to 8.2	4.0	1.6 to 9.9	3.9	1.54 to 9.9
H only	3	4	7	10	4.2	1.39 to 12	4.7	1.5 to 15	5.0	1.54 to 16
Other	30^d^	13^e^	43	11	23	10.0 to 54	23	9.7 to 55	26	10.3 to 64
TOTAL	40	39	79	143						

^a^ OR, odds ratio; CI, confidence interval; susc., susceptible. ^b^Adjusted for genotype and resistance pattern. ^c^Adjusted for age, sex, genotype, and resistance pattern. ^d^Of these 30, 12 had resistance to H and S, 1 to H, S, and E, 10 to H, R, and S, and 7 to H, R, S, E. ^e^Of these 13, 11 had resistance to H and S, and 2 to H, S, and E.

This study had limited power to detect risk factors for relapse and treatment failure, mainly because of the relatively small numbers of case-patients in those categories. Recruiting a larger number of controls could not change this, since the selection of more than two controls per case, while increasing workload, has relatively little impact on the statistical power of the analysis. However, since the association between the Beijing genotype and treatment failure or relapse was strong, the association was significant despite limited power.

Beijing genotype strains may have several selective advantages over other genotypes of *M. tuberculosis*. In many, but not all, areas where Beijing genotype strains are prevalent, this genotype is associated with resistance to antituberculosis drugs (*17*). The basis for this correlation has so far not been disclosed. However, recent findings indicated that exclusively in Beijing genotype strains, mutations are present in putative mutator genes (*18*). This finding may indicate that Beijing genotype strains have a higher ability than other strains to allow particular critical mutations in resistance genes, which enables them to acquire resistance to the drugs used in a standard treatment regimen.

This enhanced flexibility due to alterations in the DNA repair mechanism of Beijing genotype bacteria may also play a role in the interaction with the host immune defense system to deal with the less favorable conditions like exposure to oxygen and nitrogen radicals in intracellular environment. Extended research on the immunopathology caused by *M. tuberculosis* strains of different genotypes in a BALB/c mouse model has shown that most, but not all, Beijing genotype strains cause a more severe pathology, but a reduced immune response in comparison to other genotypes of *M. tuberculosis* (*19*).

If Beijing genotype strains have a selective advantage over other genotypes of *M. tuberculosis,* this may have important implications for future tuberculosis control. The enhanced capability to develop resistance and to interact with the host immune defense system may facilitate the spread of tuberculosis in Asia and in other areas. Currently, a worldwide survey is being conducted to measure the global spread of this genetically conserved group of *M. tuberculosis* strains and its association with resistance, active transmission (young age), and other factors. Although the conservation of Beijing genotype strains in Asia is highly pronounced, the conserved population structure of *M. tuberculosis* in other high-prevalence areas such as Africa also merits further research on the possible development of selective advantages.
